# The Barretos Cancer Hospital Animal Facility: Implementation and Results of a Dedicated Platform for Preclinical Oncology Models

**DOI:** 10.3390/vetsci9110636

**Published:** 2022-11-16

**Authors:** Silvia A. Teixeira, Mayara de Cassia Luzzi, Ana Carolina Baptista Moreno Martin, Terence Teixeira Duarte, Mônica de Oliveira Leal, Gustavo Ramos Teixeira, Monise Tadin Reis, Carlos Roberto Almeida Junior, Karina Santos, Matias Eliseo Melendez, Diego da Cunha Silveira Alves da Silva, Priscila Neves Bernécule, Higor Vinicius Lourenço Firmino, Ana Laura Vieira Alves, Denise Peixoto Guimarães, João Vitor Borduqui, Ana Carolina Laus, Bruna Minniti Mançano, Rui Manuel Reis

**Affiliations:** 1 Molecular Oncology Research Center, Barretos Cancer Hospital, Barretos 14784-400, São Paulo, Brazil; 2 Department of Pathology, Barretos Cancer Hospital, Barretos 14784-400, São Paulo, Brazil; 3 Barretos School of Health Sciences, Dr. Paulo Prata—FACISB, Barretos 14785-002, São Paulo, Brazil; 4 Department of Neurosurgery, Barretos Cancer Hospital, Barretos 14784-400, São Paulo, Brazil; 5 Department of Radiology, Barretos Cancer Hospital, Barretos 14784-400, São Paulo, Brazil; 6 Molecular Carcinogenesis Program, National Cancer Institute, Rio de Janeiro 20231-050, Brazil; 7 Department of Endoscopy, Barretos Cancer Hospital, Barretos 14780-000, São Paulo, Brazil; 8 Life and Health Sciences Research Institute (ICVS), School of Health Sciences, University of Minho, 4710-057 Braga, Portugal; 9 ICVS/3B’s-PT Government Associate Laboratory, 4710-057 Braga/Guimarães, Portugal

**Keywords:** PTCH1-knockout, *Apc^Min^*, xenografts, PDX, genetically engineered mouse models, personalized therapy, tumor biology

## Abstract

**Simple Summary:**

Preclinical models of Latin-American patients are scarce and urgently needed to properly translate our results, from bench to bedside, with a focus on personalized therapy. In addition, to discover more effective treatments for different cancer types we described here, an overview of the infrastructure of Barretos Cancer Hospital Animal Facility was designed to attend a multidisciplinary team and organized to perform pre-clinic in vivo models. This work describes the establishment of conventional mice models, using several commercial cancer cell lines, genetically modified mouse models that develop specific tumor types (colon cancer or brain tumor) and a platform of Brazilian patient-derived xenograft models (PDX) from patients diagnosed with cancer. These models have been used to understand cancer biology, tumor pathways and to enhance translational studies. Moreover, PDX models, which preserve cell interaction and cellular heterogeneity of parent tumors, have shown promise for identifying new biomarkers, testing panels of anticancer drug screening, and therapeutic strategies prior to clinical trials. We illustrated the establishment of a novel animal facility that fosters cancer research and preclinical studies in Brazil that will open novel avenues for studying tumor biology and tumor microenvironments to identify potential therapeutic targets, anti-cancer drugs, and personalized therapeutic approaches.

**Abstract:**

The Barretos Cancer Hospital Animal Facility (BCHAF) is a unique facility in Brazil exclusively dedicated to working with animal models for cancer research. In this article, we briefly present our modern facility and the main experiments performed, focusing on mutant strains of mice (PTCH-knockout and *Apc^Min^* mice), xenograft models, and patient-derived xenografts (PDXs). Our results show the progress and challenges in establishing these models and the need for having an appropriate representation of our cancer population to better understand tumor biology and to identify cancer biomarkers, which could be putatively targeted, allowing for personalized therapy.

## 1. Introduction

The Barretos Cancer Hospital Animal Facility (BCHAF) is a novel facility of the Molecular Oncology Research Center, of Barretos Cancer Hospital, Barretos, SP, Brazil (https://iep.hospitaldeamor.com.br, accessed on 10 May 2022). Barretos Cancer Hospital (BCH) is a reference cancer center for the prevention, diagnosis and treatment of cancer in Brazil [1,2,3,4]. With a 60-year history in 2022, BCH is a philanthropic institution that cares for predominantly underserved healthcare population referred to the Brazilian Health Public System (SUS) and free of cost. It attends to approximately 14,000 new cases per year, covering all adult and pediatric specialties. To foster translational research, an animal facility was conceived in 2017 to carry out multidisciplinary, translational research on cancer, aiming to better understand the mechanisms of cancer pathogenesis and proposing new therapeutic approaches for cancer treatment with in vivo models. It performs breeding and experimentation in SPF (specific pathogen-free) mice, genetically defined, prioritizing animal welfare and quality of research results.

Before starting to work with animal models, an Institutional Animal Care and Use Committee (IACUC) at Barretos Cancer Hospital was created in 2018. This committee comprises researchers and professionals representing different areas (biologists, veterinarians, physicians, pharmacologists, biomedicals, bioinformatics and statisticians). The IACUC analyzes all scientific projects, protocols, or animal purchase requests. The BCHAF holds a biosafety quality certificate granted by the National Technical Biosafety Commission of the Ministry of Science (CTNBio). The mice maintained at BCHAF belong to different lineages: conventional mice (C57BL/6J and Balb-C); mice who have a non-responsive immune system (thus, allowing the establishment of Patient-Derived Xenografts (PDX) or Avatar models), such as NOD Scid Gamma (NSG) and NUDE (NU/J); and genetically engineered mouse models (GEMMs), which can spontaneously manifest neoplasms, derived by modification in specific genes (e.g., *Ptch1^tm1Mps^* and *Apc^Min^* gene) [5,6,7]. Now we are using these models to establish a Brazilian platform of PDX models for pediatric and adult brain tumors and to study colon cancer. PDX models that preserve the cell interaction and cellular heterogeneity of parent tumors have furthered our understanding of tumor biology and shown promise in identifying new biomarkers, testing a panel of anticancer drug screening, and new therapeutic strategies prior to clinical trials [8,9,10,11,12,13].

This article aims to provide an overview of the infrastructure designed and multidisciplinary team organized to perform in vivo models. Moreover, we intend to describe the experiments carried out at BCHAF since its foundation and the advances achieved in this endeavor, focusing on the lineages of genetically engineered, PDX and xenograft models. The genetically engineered mice are appropriate for study and answer specific questions. Xenograft models are extensively and specially developed to validate therapeutic results obtained from in vitro experiments. In addition, the PDX model tries to mimic, in a very authentic way, the cellular and histopathology structure, the genomic profile, and the tumor heterogeneity to recapitulate the complexity of human tumors and improve our knowledge of tumor biology and the mechanisms of drug response. Moreover, these preclinical models of Latin-American patients are scarce [13,14], and are urgently needed to properly translate our results, from bench to bedside, with focus on personalized therapy and diminished cancer inequities [10,15].

## 2. Materials and Methods

### 2.1. Infrastructure

**Figure 1:**
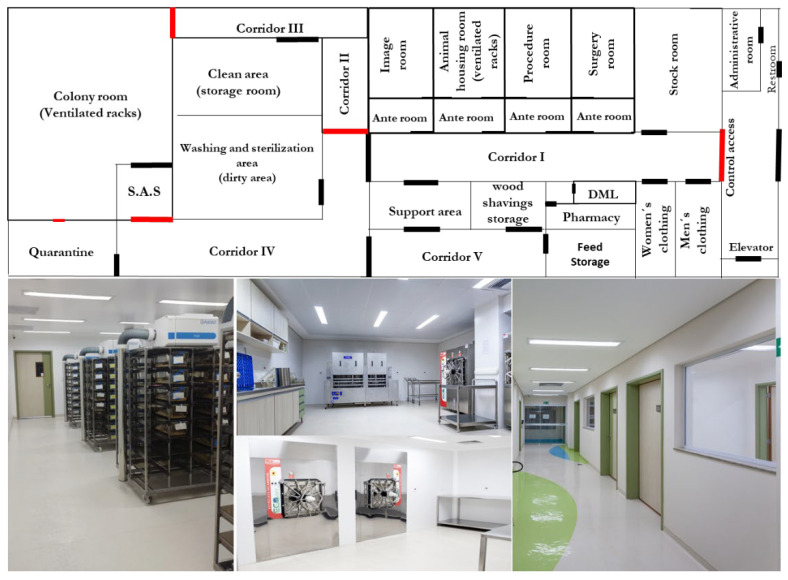
Design and structure of SPF Animal Facility (AF). The SPF AF has an area divided into different rooms. The first barrier is that personnel wearing unique disposable clothes must be dressed in a sterilized overall, mask, and cap. The rooms are equipped with racks of individually ventilated cages, autoclaves, washing cages and bottle machines. The AF area comprises animal rooms; mouse colonies; surgery room; experimental room; anteroom, storage room and corridors (clean and dirty).

**Figure 2:**
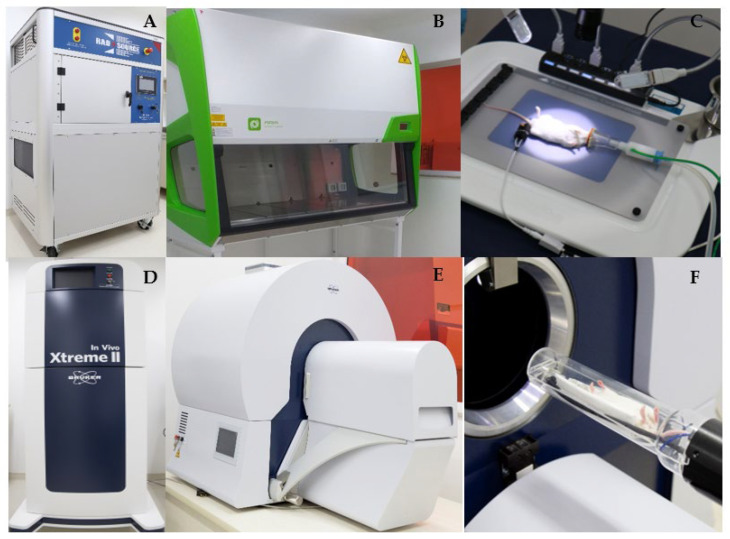
Technological equipment park. The experimental area includes modern park technology with equipment for radiotherapy [Rad-Source-2000 X-ray Irradiator] (**A**), laminar flow (**B**), a platform to perform surgical procedures with controlled temperature and accoplated anesthesia inhalator (**C**), Xtreme II (**D**) device (to analyze fluorescence, luminescence, and radioisotope), and a microtomography scanner (**E**) for small animals (**F**).

The Barretos Cancer Hospital Animal Facility (BCHAF)’ structure comprises different containment zones based on risk management and barriers “all in/all out”, to prevent contact with the AF environment and external ambiance. The entrance of BCHAF is controlled, and only trained staff, researchers and students can enter. Unique disinfected clothes and shoes must be worn. The first four rooms (Figure 1) contain all the equipment needed to develop experiments on animals. It is one of the unique animal facilities in Brazil that houses, in the same building, modern pieces of equipment, including a microtomography scanner for small animals [MicroCT (SkyScan)] (Figure 2E,F), an Xtreme II device (to analyze fluorescence, luminescence, and radioisotope) (Figure 2D), both from Bruker BioSpin Corporation, Billerica, MA, USA; a platform to perform surgical procedures with controlled temperature and inhalator anesthesia attached (Figure 2C); and radiotherapy equipment RadSource-2000 X-ray Irradiator (Rad Source Technologies, Georgia, USA) (Figure 2A). The first area of BCHAF still has rooms for storing wood shavings, feed, and medicine (Figure 1). Additionally, we have a wash and sterilization room.

A clean area is allowed for restricted staff only, and the entrance is controlled by magnetic doors opened by a personal card. In this area, we store cleaned material, food and water for mice and raise our foundation and expansion colonies of the different lineages of mice (Figure 1). In this area, a more restrictive type of clothing must be worn. Additionally, we have a quarantine room for recently arrived mice. A technical floor above AF that modulates and controls temperature and humidity 24 h to provide the mice with a safe and comfortable environment.

In addition, BCHAF has specialized and multidisciplinary staff (researchers, veterinarian, biologists, and administrative staff) that are continuously trained, aiming to develop quality animal models, ethical procedures and animal care. All this knowledge and training is given to researchers who want to develop animal experimentation in the dependencies, producing trust-worthy research results.

### 2.2. Animals

All mice models [NOD.Cg-Prkd^cscid^ Il2rg^tm1Wjl^/SzJ (NSG); NU/J (FoxN1^nu/nu^); STOCK (*Ptch1^tm1Mps^*/J); C57BL/6J; C57BL/6J-*APC^Min^*/J, C57BL/6J; Balb c/J] were purchased from Jackson’s Laboratory (USA). Mice used in these studies were housed in microisolator cages under specific pathogen-free (SPF) conditions in a dedicated mice room in the BCHAF. Mice received sterile food and water ad libitum and were maintained on a 12-h light/dark cycle. All animal experiments were performed according to protocols from IACUC at the Molecular Oncology Research Center, Barretos Cancer Hospital (Barretos, SP, Brazil), following the guidelines of the National Council for Animal Experimentation Control (CONCEA) of Brazil. All studies were carried out in compliance with ARRIVE guidelines [16]. Our animal facility staff regularly check all animal’s conditions.

### 2.3. Engineered Mice

#### 2.3.1. Genotyping

**Figure 3:**
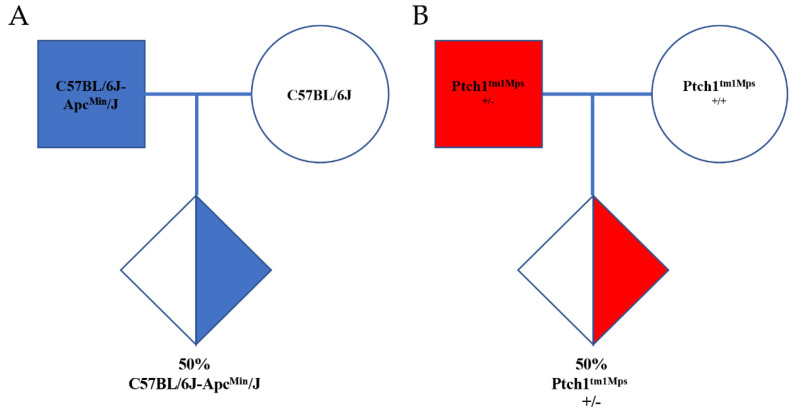
Inheritance of C57BL/6J-*Apc^Min^*/J (**A**) and STOCK *Ptch1^tm1Mps^*/6J mice (**B**). Filled symbols represent the phenotype seen in transgenic paternal and offspring, whereas half-filled symbols represent animals inheriting the transgene mutation. The percentage of a particular genotype is indicated. Diamonds show animals of both or anonymous sex.

The mice born from C57BL/6J-*Apc^Min^*/J and STOCK *Ptch^1tm1Mps^*/J colonies were genotyped to detect whether they were heterozygotic or homozygotic for the APC or PTCH1 mutation. For this, ear fragments were collected from mice aged 1 to 3 weeks and submitted to DNA extraction using Biopur Mini Spin Plus Kit (BIOPUR, Biometrix Diagnostic Ltd.a), following the manufacture’s recommendations. DNA samples were quantified by Nanodrop (ThermoFischer, Waltham, MA, USA). APC model was submitted to real-time PCR assay, according to Jackson Laboratory’s protocol (https://www.jax.org/strain/002020, accessed on 10 May 2022) (Bar Harbor, MA, USA) [6,7]. The mutant animals present a single nucleotide variant (T > A in nucleotide 2549) in APC gene. This strain was maintained by breeding between heterozygote males C57BL/6J-*Apc^Min^*/J and wild-type C57BL/6J females in our animal facility (Figure 3). APC mice were observed for the following signs of distress: abdominal edema, low-conditioned score, weakness, dehydration, hunched posture, abnormal breathing, anemia, ungrooming or piloerection, abdominal distention, decreased activity and diarrhea. When mice exhibited any of these signs of distress they were euthanized.

**Figure 4:**
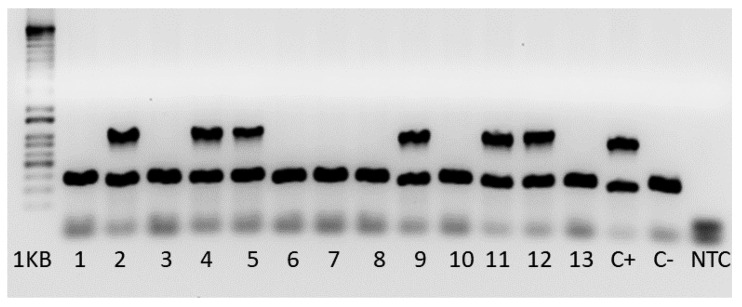
The genotyping results for *Ptcht*^m1Mps^ mice were separated by agarose gel electrophoresis. Mutant = 479 bp, Heterozygote = ~200 bp and 479 bp, Wild type = ~200 bp, C+ = Heterozygote control, C− = Wild type control.

In the PTCH model, genotypes were determined by standard PCR Assay, according to Jackson´s Laboratory (USA) protocol. Amplicons with 479 bp refer to mutant allele and amplicons with 200 bp (wild-type) allele. Samples presenting both amplicons represent heterozygous mice, and samples presenting 200 bp amplicon, represent wild-type animals (Figure 4). PTCH1 heterozygous mice were used for experimental procedures, and wild-type (wt) mice were used for control. Heterozygous mice were observed weekly for signs of disease and behavioral evidence of tumor, such as lethargy, weight loss, enlarged occipital prominence, ataxia and/or poor grooming.

All symptomatic mice were euthanized, and the tumor tissue was analyzed. Euthanasia was performed with a 3x dose of ketamine + xylazine, by intraperitoneal injection.

#### 2.3.2. Macroscopy and Histopathology

A total of 105 C57BL/6J-*Apc^Min^*/J mice were analyzed. Animal conditions and organs were analyzed to verify any abnormalities. For APC mice, intestines were washed with buffered formaldehyde (10%) to remove any residual feces. Then intestine tissue was cut longitudinally along the mesenteric line to prepare the Swiss-rolling technique [17]. The intestine was placed into cassettes and processed for paraffin embedding. Subsequently, hematoxylin and eosin (H&E) slides were prepared with 3–5 μm-thick sections.

The histopathology of small intestinal and colonic lesions were ranked following the World Health Organization’s (WHO) *Classification of Tumors of the Digestive System (5th edition)* for benign and malignant epithelial tumors and precursors of the colon and rectum. The animal lesions observed were divided into tubular adenoma, tubulovillous adenoma, intramucosal adenocarcinoma, and invasive adenocarcinoma. Moreover, adenomas were subdivided into low and high grades [18].

For PTCH mice, the brains were previously fixed with 4% buffered formalin phosphate, and later sectioned in the sagittal plane and post-fixed for at least 24 h and processed for paraffin embedding. Representative sections of mice tumors were stained with H&E and evaluated by immunohistochemistry using an automated Ventana Benchmark Ultra stainer and an Optiview detection kit (Ventana Medical System^®^). Primary antibodies used included glial fibrillary acidic protein (GFAP; clone EP672Y), OLIGO2 (clone EP112) and Ki-67 (clone 30-9). Sections were counterstained with hematoxylin. In addition, tumor slides were analyzed in a semi-quantitative manner: the positivity was classified in a two-tier system (positive or negative), independently of intensity; the cell positivity percentage was estimated based on a hotspot analysis from a single slide in each case. Non-tumoral human brain FFPE tissue was used as positive and negative external control in each reaction.

Diagnostic criteria for medulloblastoma were based on the 5th edition of the WHO Classification of Central Nervous System [19]. The brains of intact wild-type mice were also collected and processed for histological examination. The stage of neoplastic lesions in the cerebellum was classified according to progression and dissemination as an incipient, established, and invasive tumor.

### 2.4. Xenografts Model and Patient-Derived Xenograft Tumor (PDX)

The tumorigenic capacity of several commercial cell lines or primary cell culture (from patient tumor tissue) was evaluated. We have generated model mice for brain tumors [medulloblastoma (MB), glioblastoma (GBM), ependymoma (EPN), pilocytic astrocytoma (PA), neuroblastoma, high-grade glioma (HGG), low-grade glioma LGG)], lung cancer (adenocarcinoma), colon cancer (adenocarcinoma), germ cell tumor (GCT), skin cancer (melanoma) and cervix cancer.

To generate a Brazilian PDX model, fresh tumor tissues were collected from patients undergoing surgery. For this, patients diagnosed with brain tumor (pediatric and adult) or colon cancer (adult) were included in the study. The tumor tissue was transferred to the BCHAF and was used to establish primary cell culture (2D/3D) [14], a biorepository (cryo-preserved tumor tissue and primary culture) [2] and PDX models. We developed a specific protocol for the PDX model to obtain fresh tumor tissue from solid tumors. The PDX models were generated by implanting tissue fragments or primary cells from surgical resections. The specimens were enriched with Matrigel and injected/implanted into immunocompromised mice to produce a subcutaneous PDX model.

The human clinicopathological data were collected from medical records. Patients signed informed consent forms, and all experimental protocols were approved by the Local Ethics Committee, with the references 4.667.471 and 4.703.892, performed under the Guidelines and Standards Regulation for Research Involving Human Beings.

#### 2.4.1. Establishment of Subcutaneous Tumors

For the xenograft model, commercial cell lines of several tumor type (SNC, colon, cervix, lung, embryonic cells) were used. A total of 1 × 10^6^ to 1.0 × 10^7^ cells/100 μL in HBSS (Hank’s Balanced Salt Solutions) were resuspended in Matrigel^®^ and were implanted subcutaneously (SC) into the right flank of NSG or NUDE mice, aged 6–8 weeks. The PDX models were generated using primary cell culture (1 × 10^6^), or fresh surgical tumor tissue, collected from patients undergoing surgery at Barretos Cancer Hospital. The tumor tissue was sliced into small fragments (1–3 mm) according to tumor type (brain tumor or colon cancer) [20] and engrafted subcutaneously in NUDE or NSG mice. All mice were maintained under specific pathogen-free (SPF) conditions and received sterile food and water ad libitum. Animals were monitored for signs of morbidity and tumor burden, and weights were recorded three times per week. Representative sections of mice tumors (subcutaneous) were stained with H&E. To validate the efficacy of drugs and to prove in vitro results, animal models with a brain tumor, lung cancer, or cervix cancer were treated with specific drugs. Overall survival and tumor volume after treatment were analyzed. Then, when tumors reached a volume of 800 to 1500 mm^3^, mice were euthanized. The engrafted tumor was removed aseptically and preserved in formalin for histopathology diagnosis and immunohistochemistry and cryopreserved in our Institutional Biobank for further molecular analysis and serial transplant (in the case of the PDX model).

**Figure 5:**
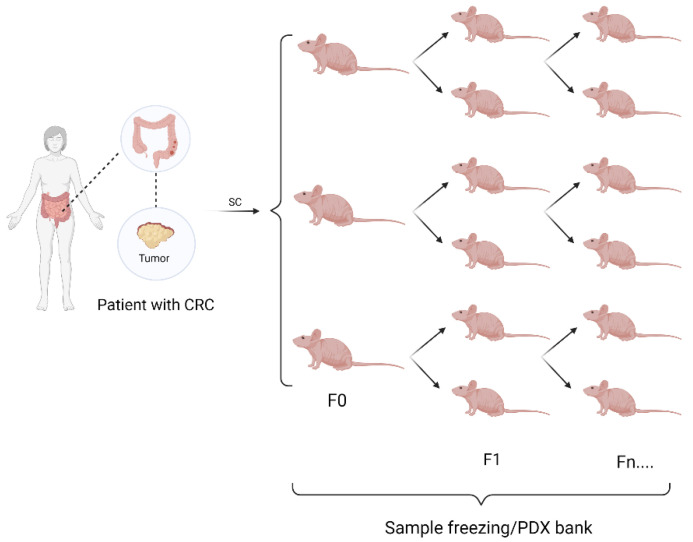
Generation of solid-tumor PDX models. Establishment of the animal model derived from a human colon tumor fragment. Small fragments are implanted in the subcutaneous tissue of three animals (F0). Next, the tumors that develop are reimplanted in another two animals (F1), successively (Fn…) All fragment samples are freezing in PDX banks.

To establish the PDX model, several passages of transplanted mice are necessary, and the first group of mice transplanted with primary culture or human tumor tissue is designated as first passage 0 (F0). Implantation of tissue samples harvested in later passages were designated as F1, F2, … Fn (Figure 5). We have successfully serial-transplanted subcutaneous tumors up to passage 4, the highest passage number used to minimize genetic drift.

After the tumor draft, the animal was monitored weekly, and tumor volume was measured three times per week with a caliper and calculated using the formula: VT = Dxd2/2 where D represents the longest diameter and d the shortest diameters of the tumor [21,22]. When the experiment involved treatment, we established that doses that resulted in mortality or a body weight loss greater than 20% were considered toxic. Antitumor effects were quantified as relative tumor volume in treated groups compared with the control group. All mice were maintained under SPF conditions and received sterile food and water ad libitum.

#### 2.4.2. Orthotopic Xenograft Tumors (Brain Tumor)

The first orthotopic mice models we established were brain tumors (glioblastoma and medulloblastoma). Orthotopic models were generated using a GBM commercial cell line (U87), acquired from the American Type Culture (ATCC), and primary cell HCB151 established at Barretos Cancer Hospital as previously described [23,24,25,26].

The medulloblastoma models were generated using a DAOY commercial cell line, purchased from ATCC. To establish medulloblastoma models 3 × 10^5^ DAOY cell suspension was aspirated into a 10 μL-attached Hamilton^®^ syringe and injected into the 2 mm-hole posterior to the lambda suture, 2 mm deep, according to guidelines suggested by Gholamin et al. [27]. To the orthotopic GBM model, a total of 1.5 × 10^5^ U87 expressing luciferin were implanted intracranially in the striatum of NUDE mice, aged 8 weeks [28]. At the end of the procedure, the animals were treated intraperitoneal (IP) with the analgesic Meloxicam (2 mg/kg) every 24 h and Tramadol (12.5 mg/kg) every 8 h, for 3 consecutive days. Animals were monitored for signs of morbidity, and weights were recorded three times per week after surgery. The mice were treated, on day 8 after surgery, with sulphonamide, as previously described [23]. The drug was injected intraperitoneally, three times a week for 3 weeks at a 50 mg/kg dose [28]. The mouse was irradiated individually after being anesthetized with 10% ketamine (80 mg/kg) and 2% xylazine (10 mg/kg). In order to more accurately model treatment in which radiation is given in fractions and localized to the tumor region, we used image-guided fractionated irradiation on the Linear accelerator (True Beam STX, Varian Medical Systems). The brain was imaged to confirm tumor burden. Mice were assigned to 4 groups of treatment. The mice received six applications of 7Gy, using the standard fractionation technique (30x2Gy/fractions) in human treatment. The fractions were applied with an energy of 6 MeV and a dose-rate of 600 MU/Min. Mice were re-imaged after two and four weeks following radiation. Control animals were only treated with vehicle solution. Animals were monitored for signs of morbidity and weight. Tumor growths were monitored until their survival endpoint. All mice were anesthetized and transcardially perfused with phosphate-buffered saline, followed by 4% (p/v) paraformaldehyde. The brain was removed, fixed in the same solution for 24 h at 4 °C and harvested for histologic analysis. Representative sections of mice tumors (subcutaneous and orthotopic) were stained with hematoxylin and eosin (H&E). The neuropathologists reviewed all H&E slides.

### 2.5. Imaging of Xenograft Tumor

Tumor growth was monitored during all experiments using scanning equipment for small animals, MicroCT (SkyScan, Bruker) and Xtreme II (Bruker BioSpin Corporation, MA, USA) to analyze fluorescence, luminescence, and radioisotope). Microtomography analysis was shown to illustrate tumor growth. In addition, the design of the AF, the acquisition of equipment for treatment and for capturing images, the individualized and personalized training, and the establishment and characterization of experimental models are essential factors that contribute to animal care and well-being and to the reduction of the number of animals used in the research. Therapeutic tests performed previously in vitro also contribute to a better experimental design and to the validation of therapeutic compounds for in vivo models.

## 3. Results

### 3.1. Genetically Engineered APC Mice

**Figure 6:**
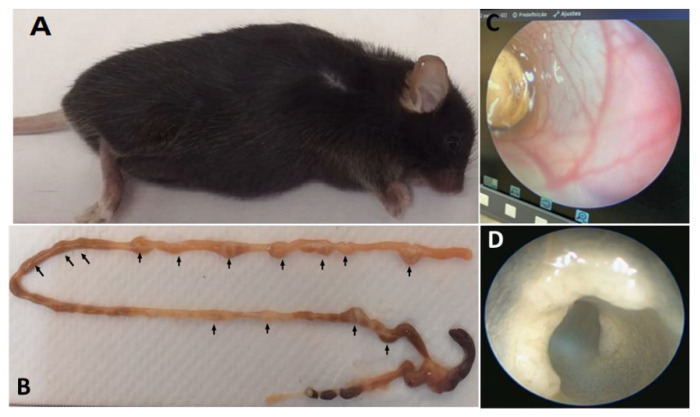
Figure representing heterozygous mouse of the C57BL/6J-*Apc^Min^*/J. *(***A**) C57BL/6J-*Apc^Min^*/J mouse heterozygote with intestinal lesions; (**B**) Polypoid lesions along of the intestine (arrows); (**C**,**D**) Illustrative figure obtained using Coloview Mainz-Storz equipment to identify intestinal lesions in C57BL/6J-*Apc^Min^*/J mice.

**Figure 7:**
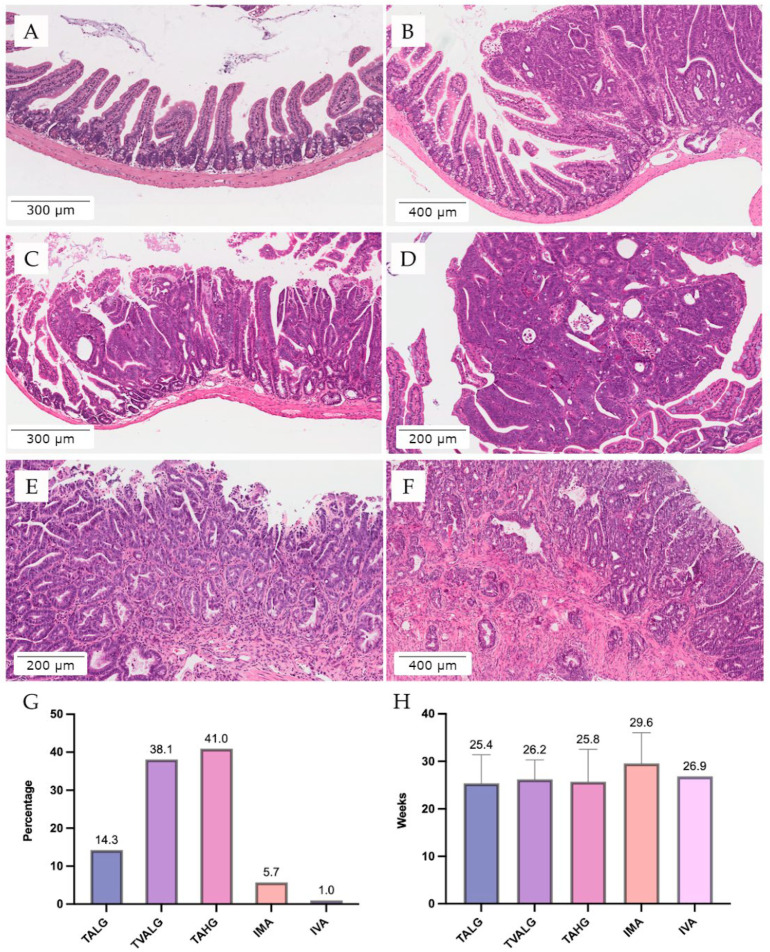
Representative colon lesions from C57BL/6J-*Apc^Min^*/J animal mouse model. (**A**) Normal small intestine (100X magnification). (**B**) Tubular adenoma, low-grade (TSLG) with herniation (100X magnification). (**C**) Tubulovillous adenoma, low-grade (TVALG) with a single layer of normal epithelium. (**D**) Tubular adenoma, high-grade (TAHG) with Paneth cells (100X magnification). (**E**) Intramucosal adenocarcinoma (IMA, 100X magnification). (**F**) Invasive adenocarcinoma (IVA, 100X magnification). Percentage of mice (**G**) and average life (**H**) of the different types of colon lesions.

Regarding our C57BL/6J-*Apc^Min^*/J colony, we generated, between 2019 and 2021, 401 mice. Among these mice, 45.1% and 54.9% were female and male, respectively. Considering the genotypes, 56.5 % were C57BL/6J, and 43.5% were C57BL/6J-*Apc^Min^*/J, as described in Table 1. Gender percentages from each genotype were as expected for a mendelian ratio. Post-mortem analyses were discarded due to tissue degradation; therefore around 60% of our C57BL/6J-*Apc^Min^*/J mice were euthanized, and their small intestine slides were analyzed by an expert pathologist (Figure 6 and Figure 7).

Among all mice bred using C57BL/6J-*Apc^Min^*/J males and C57BL/6J females, 401 were born from these animals; 220 were male and 181 were female. Therefore, from the total of 401 mice born, 43.5% (which corresponds to 175 mice) had the expected genotype for APC mutation (heterozygous–C57BL/6J-*Apc^Min^*/J) and 56.5% were C57BL/6J (226 mice). The C57BL/6J mice were used to keep the colony, the C57BL/6J-*Apc^Min^*/J male was used to keep the colony and both genders were used to analyze the development of adenomas. From the C57BL/6J-*Apc^Min^*/J mice, we were able to analyze 105 mice that were subdivided into five categories: tubular adenoma low-grade (TALG), tubulovillous adenoma low-grade (TVALG), tubular adenoma high-grade (TAHG), intramucosal adenocarcinoma (IMA), and invasive adenocarcinoma. The number of each category is described in Table 2.

Most C57BL/6J-*Apc^Min^*/J cancer lesions are localized in the small intestines and are usually only visualized and analyzed after euthanasia (Figure 6A,B). Therefore, methods and techniques that provide the facility to visualize these lesions prior to euthanasia are essential to monitoring the lesions’ differentiation and organizing possible experiments (Figure 6C,D). Thus, we tested a piece colonoscopy equipment Coloview Mainz-Storz (Karl Storz SE & Co. KG, Tuttlingen, Germany) to visualize and identify the lesions from the animals from the rectum through the colon and small intestines. This animal model usually takes longer to develop lesions; these lesions are typically less aggressive compared to clinical features found in human patients.

Regarding the lesions found in our C57BL/6J-*Apc^Min^*/J animals, we could differentiate into tubular adenoma characterized by a preserved typical crypt architecture. The epithelium was enlarged with hyperchromatic nuclei, nuclear stratification, and loss of polarity. A small villous component (<25%) was accepted. Tubulovillous adenoma had the same characteristics as tubular adenoma; however, villous structures were between 25% and 75%. The adenomas were further graduated into a two-tiered system of dysplasia: low- (LG) and high-grade (HG). HG is characterized by marked complex glandular crowding and cribriform architecture accompanied by cytological features with markedly enlarged nuclei and prominent nucleoli. Tubular adenoma, low-grade (Figure 7B), and Tubulovillous adenoma, low-grade (Figure 7C), were found in 14.3% and 38.1% of C57BL/6J-*Apc^Min^*/J mice, respectively (Figure 7G). Tubular adenoma high grade was presented in 41% of our APC colony (Figure 7D,G).

Intramucosal adenocarcinoma is considered a malignant epithelial tumor with glandular differentiation restricted to mucosal lamina propria and was observed in 5.7% of our mice (Figure 7E,G). Invasive adenocarcinoma, characterized as a malignant epithelial tumor with glandular differentiation and invasion through the muscularis mucosae into the submucosa, was found in only one mouse, representing 1% (Figure 7F,G).

Additional features on histopathological analysis included a single layer of normal epithelium overlying the adenomas (Figure 7C), adenomatous mucosa herniation (Figure 7B), and the presence of other cell types than absorptive cells such as goblet cells and Paneth cells (Figure 7D). Interestingly, herniation is a vital fall that could lead to a misdiagnosed invasive adenocarcinoma.

### 3.2. PTCH1^+/−^ One-Copy Deletion Promotes MB Development in Mice

**Figure 8:**
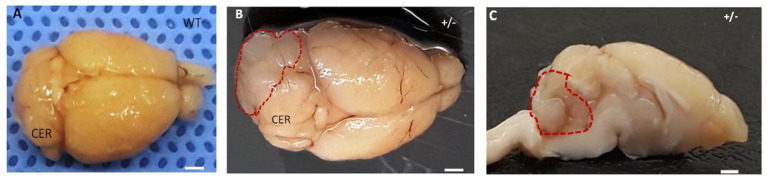
Deletion of the PTCH1 allele leads to medulloblastoma. Illustrative photography of brain mice. (**A**) Brain of wild-type (WT) mice; (**B**,**C**) PTCH1^+/−^ brains illustrating tumor (outlined in red). (**C**) Brain in sagittal section illustrating hydrocephaly and cerebellar tumor. Bars = 1.8 mm.

Of fifty-two PTCH1 heterozygous mice evaluated, twelve (12/52, 23,07%) showed enlargement of the cerebellum (Figure 8). These animals displayed apparent symptoms of ataxia and diminished activity. The presented symptoms at a median age of 30.3 (± 8.4) weeks, and hydrocephalus was detected in six (6/12, 50,0%) (Figure 8C). Tumors were more frequent in male (n = 8) than female (n = 4). The brains affected by medulloblastomas were easily recognized by their abnormal cerebellar growth, and the tumors appeared as mass lesions compressing neighboring normal-appearing cerebellar tissue (Figure 8B,C).

**Figure 9:**
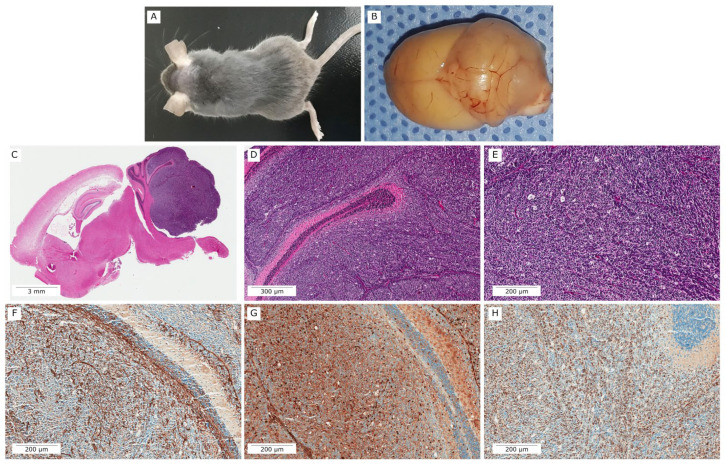
Representative images of a large medulloblastoma on a PTCH1^+/−^ mouse. (**A**) PTCH1^+/−^ mouse that developed cerebellar lesion. (**B**) Cerebellar enlargement with increased vascularity and focal areas of hemorrhage. (**C**) Whole-slide scanning of mid-parasagittal section revealing large tumor on cerebellum. Medulloblastoma extending on cerebellar folia ((**D**), 50X magnification) with classical morphology (**E**). Immunostaining for GFAP (**F**) and OLIG2 (**G**) revealed entrapped glial cells. (**H**) High Ki-67 index (30–40%). (**E**–**G**) 100X magnification.

At necropsy, the brains containing medulloblastoma lesions were recognized by their abnormal cerebellar development (cerebellar dysplasia). The PTCH1^+/−^ mouse that developed medulloblastoma tumors exhibited dense cerebellar lesions that compress adjacent normal tissue. The cerebellum´s swelling and cerebellar foliation blurring were macroscopically observed, and tumor cells ranging from the midline to lateral locations and extended over multiple folia (Figure 9A–C). Small clusters of tumor cells in the granular layer (GL) were classified as incipient tumors, and dense masses of cerebellar lesions with increased areas of neovascularization were classified as advanced tumors (Figure 9B).

Histopathological analysis revealed high-cellular tumors, mainly composed of cells with high nuclei-cytoplasm ratios and small dark nuclei, distributed in sheets along with the cerebellum white matter (Figure 9C–E). High mitotic index and apoptotic figures were identified among tumor cells, mitotic count ranged from 6 to 22 mitosis in 2.38 mm^2^. All cases (12/12, 100%) were classified as classical medulloblastomas. None had desmoplastic/nodular nor large cell features. Immunostaining for GFAP and OLIG2 revealed entrapped reactive glial cells among all tumor samples (Figure 9F,G). Ki-67 ranged from 20% to 50% on hotspots (Figure 9H).

### 3.3. Subcutaneous Xenograft In Vivo Model

**Figure 10:**
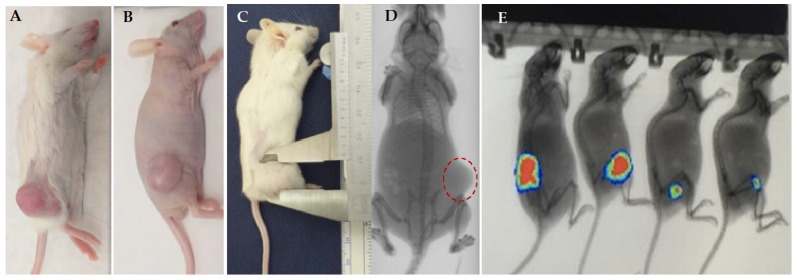
Representative xenograft models generated at BCHAF using different tumor type and cells (primary cell culture or commercial tumor cells). All tumor cells were inoculated subcutaneously, in the right flank, of NUDE (**B**,**D**) or NSG mice (**A**,**C**,**E**). (**A**–**E**) Illustrative image representative of tumor type of animal in different phases of tumor development. Images representative of (**A**,**E**) brain tumor (glioblastoma); (**B**,**D**) lung cancer; (**C**) Tumor measure with a caliper (brain tumor); the presence of tumor mass was documented using a microtomography (Micro-CT, Bruker) (**D**) or Xtreme II (Bruker) (**E**).

The subcutaneous xenograft model was established in immunocompromised mice [NSG (Figure 10A,C) or NUDE (Figure 10B)] using several cell lines that were representative of different cancer types (brain tumor, cervix cancer, embryonic tumor, skin cancer and lung cancer). To analyze the tumorigenic capacity of the cells, two animal strains were used according to cell type, cell concentration and enriched Matrigel^®^. The time to generate tumors ranged from 2 weeks to 8 months, with the average time for most strains being two months. The tumor growth was measured with a caliper (Figure 10C) and imaged using MicroCt (Bruker) (Figure 10D) or monitored by Xtreme II (Bruker) when the cells expressed bioluminescent, fluorescent, or radioisotope markers (Figure 10E). The tumor growth or therapeutic effect of treatment were imaged by Xtreme II.

**Figure 11:**
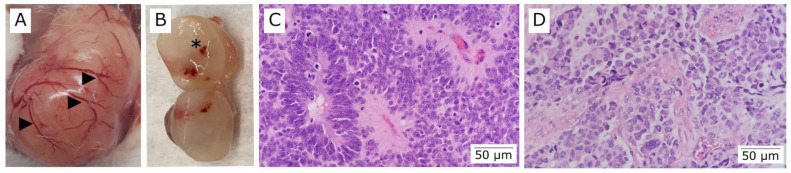
Representative image of tumor generated in the subcutaneous of xenograft models. Photography of tumor generated in the subcutaneous immunocompromised mice. (**A**,**D**) High Grade Glioma (HGG). (**A**) The tumor exhibited intense neovascularization and vases ramification (arrows) and (**D**) Neuroepithelial tumor with glial differentiation and astrocytic morphology. Tumor exhibits high mitotic activity and microvascular proliferation. (**B**,**C**) Ependymoma. (**B**) Longitudinally sectioned tumor illustrating presence of solid tumor (stars), small area of hemorrhage, and necrosis and (**C**) tumor with glial differentiation. All tumors were analyzed using optical microscopy in vivo. Photomicrographs of tumors (H&E stain) (400X magnification).

At the end of the experiments, the tumors were collected and analyzed macroscopically and by optical microscopy. The tumor generally exhibits neovascularization (Figure 11A) and hemorrhagic and calcification areas (Figure 11B). The histopathologic characteristics were analyzed by microscopy to classify tumor-grade tumors according to the presence of mitosis, necrosis area and neovascularization, and to identify the effect of treatment of tumor cells and tumor evolution (Figure 11C,D).

### 3.4. Orthotopic Model

**Figure 12:**
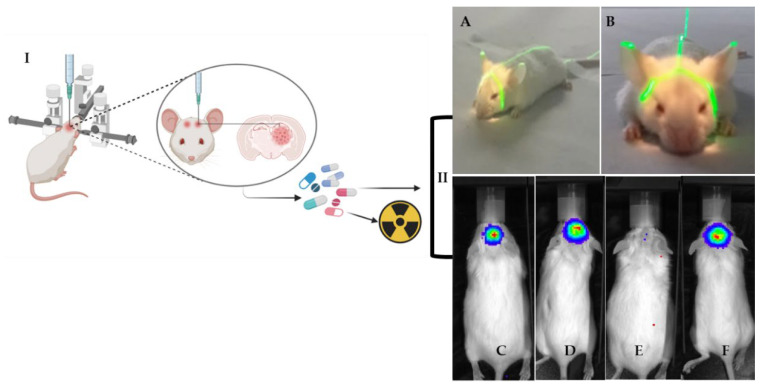
**I.** Drawing of orthotopic implantation of brain tumor cells in immunocompromised mice to therapeutic treatment (drug and radiotherapy). **II.** Illustrative images of orthotopic mouse models to assess treatment response. (**A**,**B**) Positioning the animal on the linear accelerator to radiotherapy treatment. The field light shows a defined area of 2 × 2 cm in which the radiation was directed to the tumor area (craniocaudal view). (**C**–**F**): Luciferase bioluminescence for: (**C**) Control (mouse treated with the vehicle); (**D**) Drug (mouse treated only with drug); (**E**) Radiotherapy + Drug (combination of treatments) and (**F**) Radiotherapy (mouse treated only with radiotherapy).

Subsequently, we developed the orthotopic xenograft model. Orthotopic implantation models are considered superior to the subcutaneous xenograft model because the tumors grow under the influence of the local organ-specific microenvironment. The orthotopic implantation models’ cells are surgically implanted in the mouse in the same tissue of origin (Figure 12) as into the brain as documented here.

To establish orthotopic models and evaluate the therapeutic effect of drugs that pass by the blood–brain barrier (BBB), we used U87 GBM cells and a sulphonamide (Indisulam, 50 mg/kg), previously studied by our group, to sensitize GBM cells to the radiotherapy treatment [23]. All of mice developed glioma xenografts. The athymic nude mice were treated alone or associated with radiotherapy, the standard of care for glioblastoma patients. These treatments did not significantly change body weight or cause any observable toxicity (data not shown). The results indicated that pre-treatment with the drug sensitized the tumor mice to radiotherapy and reduced tumor volume (Figure 12E).

### 3.5. Patient-Derived Xenograft (PDX) Model

**Figure 13:**
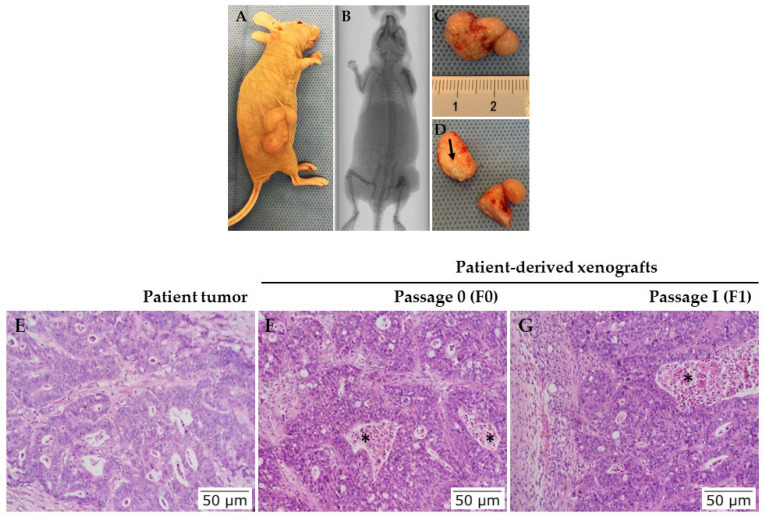
Establishment of patient-derived xenograft (PDX) models. (**A**) Representative image of colorectal tumor generated in the subcutaneous of NUDE mice. The tumor exhibited intense neovascularization and vases ramification (arrows). (**B**) The presence of tumor mass was documented using microtomography (Micro-CT, Bruker). (**C**) The tumor was analyzed macroscopically and processed for histological analysis. (**D**) Longitudinally sectioned tumor illustrating presence of necrosis area (arrow). (**E**) H&E staining of the patient’s tumor after surgical resection (200X magnification). (**F**,**G**) H&E staining of tumors F0 and F1, generated from subcutaneous implantation of tumor fragments in NUDE mice (200X magnification). Dirty necrosis areas (stars).

A PDX model was successfully generated by implanting tumor tissue/tumor cells into the male or female flanks of NUDE or NSG mice (Figure 13A). Tumors were removed, dissociated, and reimplanted in successive recipient animals. The time taken to generate PDX tumors ranged from 2 weeks to 8 months. Once the individual PDXs grow in the mice, they are expanded for banking of tumors and undergo molecular, cellular, and histological characterization concurrently with the patient tumor. A PDX model was successfully generated by implanting tumor tissue/tumor cells into the male or female flanks of NUDE or NSG mice (Figure 13A). Tumors were removed, dissociated, and reimplanted in successive recipient animals. Histological assessment of the patient tumor and matched PDX tumors was performed to characterize the model and ensure that the PDX faithfully recapitulated the original patient tumor after different generations. We performed H&E staining on PDX/patient tumor pairs. The success rate for establishing PDX colon cancer models is 50%. 

Once the tumor reached a volume of ~1500 mm^3^, it was collected and analyzed macroscopically (vascularization, hemorrhage area, presence of metastasis in other organs and necrosis area) (Figure 13C,D) and histologically through optical microscopy using a colorectal cancer pathologist (Figure 13E–G). H&E staining classified the type and grade of the tumors. PDX models were evaluated histologically and showed a good correlation between the patient’s tumor characteristics. Both were identified as adenocarcinoma, NOS, and moderately differentiated (low-grade) [29]. In the PDX-modelled tumor, dirty necrosis was more evident concerning the patient’s tumor. However, this pattern is considered standard in colorectal cancer (Figure 13F,G) [30,31].

Finally, our xenograft model replicated the histopathological features of the original tumor, demonstrating that in vivo transplantation models may facilitate biological and future preclinical studies.

## 4. Discussion

The Barretos Cancer Hospital Animal Facility (BCHAF) is a modern and comprehensive facility, which was designed for cancer translational research and preclinical studies. It was built to accommodate exquisitely controlled environments for the care and maintenance of breeding and experimentation of mice [32]. The BCHAF, which is located at the Molecular Oncology Research Center, where other in vitro and genomic platforms are in place, together with the institutional Biobank, enables the development and application of research in the translational field.

We started the establishment of genetically engineered and/or carcinogen-induced mice models, which are alternative immunocompetent models [31]. These models have been used to understand cancer biology, tumor pathways and stages of tumor development, and to enhance translational studies with more accurate models harboring relevant mutations that develop during human tumorigenesis. In addition, the second model established at BCHAF was the GEMMs, which develop intestinal adenomas (C57BL/6J-*Apc^Min^*/J) or brain tumors (STOCK *Ptch^1tm1Mps^*/J).

The C57BL/6J-*Apc^Min^* mice are GEMMs, by a chemically induced mutation, known to spontaneously develop intestinal adenomas [6,7]. Although they are widely used in experimentation worldwide, it is necessary to understand the colonies individually. This strain shows good reproductive indices and both heterozygous male and female mice can develop intestinal polyps. Therefore, we initially determined when lesions start to develop in the small intestinal mice because of when animals manifest symptoms [33]. To analyze these symptoms, mice were evaluated each day by animal facility staff. From 175 heterozygote mice, 105 developed the phenotype with the presence of lesions; however, 70 mice that did not develop the phenotype or any signs of distress were kept until they were considered elderly. Of these 70 mice, only four, despite not showing any symptoms of illness, died overnight, and it was not possible to analyze the intestine. The rest of these mice did not present macroscopic lesions; therefore, they were not analyzed for histology. Additionally, a colonoscopy exam or other detection method is necessary to evaluate the presence of lesions in APC mice, especially when therapeutic or prevention treatments are being tested.

Regarding the engineered model for generating a specific brain tumor, medulloblastoma subtype SHH (Sonic Hedgehog) was selected [34,35]. Since the discovery of the SHH pathway aberrates activation in cancers, the single-allele PTCH1-knockout mouse model has influenced our understanding of tumor development and is a valuable model that recaps the development of SHH-activated tumors [5,35,36]. Therefore, the most challenging part of working with this strain is determining when the animals start to develop tumors. According to the literature, the disease starts when animals are 120–150 days old [6]; however, for the researcher, knowing the exact moment when this process starts is crucial. In agreement with the demand for directed therapies, our animal facility is also producing the SHH MB mouse model with higher medulloblastoma by conditional deletion of PTCH1 in cerebellar granule cell precursors (Math1). These tumors arise early and mimic childhood SHH MB [37]. Additionally, using a versatile strategy for profiling tumor-associated astrocytes from medulloblastoma–SHH, our facility is crossing these medulloblastoma-prone mice with bacterial artificial chromosome-translating ribosome affinity purification (bacTRAP) mice in order to access mRNAs from specific cell types of childhood SHH MB mice models [38,39]. The insights from these results should help further risk-stratification approaches and will open new therapeutic strategies that rely on genes specifically expressed in these tumor-associated cell types [37].

For the establishment of a subcutaneous and orthotopic xenograft model, we first selected two commercial brain tumor cell lines [U87 (glioblastoma) and DAOY (medulloblastoma) [40,41,42], well-characterized commercial cell lines and a primary culture HCB151 (obtained from a patient with diagnostic of GBM at BCH) [43]. As previously described by our group and others, these commercial cell lines took only three weeks to develop tumors (glioblastoma or medulloblastoma) [23]. The time to develop brain tumor using U87 GBM cell line and the radio-sensitizing effect of Indisulam is in accordance with our previous in vitro results [23]. Moreover, the results suggest that the orthotopic model using U87 was implanted at our animal facility and could be proposed to evaluate the therapeutic effects of new compounds or drug associations. However, the time to confirm in vivo tumorigenic capacity of fresh tissue obtained from surgery, or primary culture cells, extended from 1 month to 12 months. In addition, several in vivo models were established using cell lines (commercial or primary cells) from different human cancer types (lung, colon, cervix, skin, brain and embryonic tumor). These results demonstrated the relevance of setting the time to assess the tumorigenic capacity of tumor cells and to consider establishing in vivo tumor models. Depending on histology and growth rate, different tumor types might grow preferentially in different strains, as a highly aggressive tumor that is fast-growing in humans may grow fast in NUDE mice. However, immunogenic tumors may grow better in more severely immunocompromised mice [44]. In addition, over the years, mouse models have evolved from simple cell line-based heterotopic and orthotopic xenografts in immunocompromised mice to more complex GEMMs involving multigene manipulations [45]. In addition to comparing results obtained with the engineered mice to understand tumor biology better and to have a representative model with heterogeneity and microenvironment detected in human tumors, we started to work with the PDX/Avatar model.

To capture the complexity and diversity of solid tumors or to establish new models of recurrent disease, especially pediatric brain tumors and colon cancer, here we developed a protocol to produce PDX at diagnosis and recurrence. The PDX models of colon cancer and brain tumors were successfully generated on our AF and morphologically faithfully recapitulated the original patient tumor after different generations. The PDX model preserves cell interaction and has furthered the understanding of tumor biology, tumor genetic evolution and tumor pathobiology, and shown promise for identifying new biomarkers, as well as offering a tool for developing anticancer therapies and personalized medicine for patients with cancer, and has contributed to the poor outcomes of numerous clinical trials [20,45,46,47].

Patient-derived xenograft models, established by implanting fresh tumor tissue from patients into immunocompromised mice (SCID/NUDE) are the gold standard in cancer research for understanding disease progression and preclinical testing of new therapies [45,48,49]. These models maintain close similarity with the original patient tumor, preserving the morphological and genetic heterogeneity of human cancer and recapitulating the responses in the clinic, representing an emerging and powerful tool and a significant advance in preclinical testing [50]. PDX models are currently used in the preclinical therapeutic screening of drugs for several cancers [51,52,53,54], and in tests of chemotherapeutic drugs, there could be good correlations between PDX models and human outcomes. In addition, the implantation of tumor cells into the organ of origin (orthotopically) allows organotypic interactions between tumor cells and the surrounding stroma [55,56]. The orthotopic models are considered superior to the subcutaneous xenograft model because the tumors grow under the influence of the local organ-specific microenvironment and have been widely used for the optimization of target therapies and preclinical evaluation of therapeutic modalities [44]. In addition, PDX has emerged as a valuable model for many cancers and has contributed significantly to understanding tumor biology [57]. This model added value in narrowing the preclinical and clinical research gap to develop stratified therapies [55].

In order to discover more effective treatments and improve patient survival rates, are necessary models through which it will be possible to identify potential molecular targets and then test appropriate therapeutics preclinically. In the same way, in vitro models are helpful; however, there is a limit to their translational utility, and indeed variation in culture methods can significantly impact gene expression and drug responses. However, an alternative to promoting precision medicine or personalized medicine is the establishment of an animal model with a humanized immune system (hu-PDX model) [58]. The PDX model, which recapitulates the biology, heterogeneity and tumor microenvironment of tumor patient, have been proposed for the development of humanized models. To generate hu-PDX and reconstruct the human immune system, immunodeficient mice were engrafted with functional human cells (hematopoietic cells or lymphocytes) or tissue [59,60]. These models modulate the interactions between immune components and tumors of human origin, provide a preclinical evaluation of onco-immunotherapies and are used to the development of anti-cancer drugs, co-clinical trials, personalized medicine and PDX biobanks; although there are some limitations to working with these models. In the future, it will be essential to focus on hu-PDX to study human disease, tumor–immune system interactions, and treatment combinations to implement personalized medicine and minimize failures in clinical trials [58,59].

## 5. Conclusions

We illustrated the establishment of a novel animal facility to foster cancer research and preclinical studies in Brazil that captures the admixture of its population. Moreover, we reported the characterization of GEMMs and the successful establishment of xenograft models for several tumor types, GEMMs, and PDX models of Brazilian brain tumor and colon cancer that will open novel avenues to study tumor biology and the tumor microenvironment and identify potential therapeutic targets, anti-cancer drugs and novel personalized therapeutic approaches.

## Figures and Tables

**Table 1:** Colony data of C57BL/6J-*Apc^Min^*/J animal model between 2019 and 2021.

	Animal Facility (AF)	
Male	220 (54.9%)	
Female	181 (45.1%)	
	Homozygous C57BL/6J	Heterozygous C57BL/6J-*Apc^Min/+^*
Mice %	56.5	43.5
Male mice %	56.2	52.9
Female mice %	43.8	47.1
Average life (weeks)	-	26.12 ± 5.7

**Table 2:** Number of C57BL/6J-*Apc^Min^*/J mice analyzed regarding lesion types.

	Lesion Type	TALG	TVALG	TAHG	IMA	IVA
Mice Number						
105		15	40	43	6	1

## Data Availability

The original contributions presented in the study are included in the article. Further inquiries can be directed to the corresponding author.
